# The Hospital Nursing Supplement

**Published:** 1896-01-04

**Authors:** 


"The Hospital" Nursing Mirror.

Being the Extra Nursing Supplement of "The Hospital" Newspaper.

[Contributions for this Supplement should be addressed to the Editor, The Hospital, 428, Strand, London, W.C., and should have the word "Nursing" plainly written in left-hand top corner of the envelope.]

### News from the Nursing World.

### FROM YEAR TO YEAR.

When the annual entertainments at hospitals and infirmaries attract from the public special attention to their nursing departments, improvements are generally found to have taken place during the interval since the last anniversary. Sometimes the uniform has been changed or modified, or the nurses' quarters have been increased, and a pleasant sitting-room or piano secured for their use. Again, it may be a library which has been instituted, or a tennis lawn laid down. There is plenty of scope for committees and other friends of nurses to mark each New Year by some practical improvement, and the annual entertainment furnishes a valuable opportunity of comparing notes on the progress of sister institutions.

### ROYAL NATIONAL PENSION FUND FOR NURSES.

Policy-holders and our readers generally may be intested to hear that the year 1895 has been a record one for this fund. Altogether 915 new proposals have been received, and 821 new policies have been issued, against 610 proposals and 563 new policies granted in 1894. The business during 1895 represents an increase of over 44 per cent. compared with the previous year, a fact upon which we congratulate the council, the secretary, and all who are interested in the fund. We wish them all a bright and prosperous new year.

### THE CORNEY GRAIN COT.

The sum of money raised for the endowment of a cot at the Great Ormond Street Children's Hospital, in memory of Mr. Corney Grain, has proved sufficient to provide also for an annual distribution of toys to the small inmates. The first of these gifts were handed to the little ones last week on the occasion of their Christmas treat.

### ROYAL FREE STUDENTS AND NURSES.

A great change has taken place since last year in the accommodation provided for the women students at the Royal Free Hospital, and now it is to be hoped that some further improvements in the nurses' quarters may receive consideration. The adoption of a pretty and neat style of cap by the nursing staff has received complimentary notice from many visitors.

### EARNINGS OF PRIVATE NURSES.

It is announced by a correspondent in the local Press that the Lady Superintendent of the Royal Infirmary, Hull, will be glad to receive applications for nurses. With the approval of the board she has organised a private nursing branch in connection with the infirmary, and hopes thus to retain the services of some of the probationers who have completed a course of three years' training in the infirmary. In addition to the obvious advantage to the neighbourhood of securing a constant supply of skilled nurses for private cases, the writer of the letter in question also suggests that the income of the institution will be increased by the scheme; but we fail to see how that can rightly be expected. If the private nursing branch becomes entirely self-supporting, when the funds of the infirmary have been recouped for such expense and risk as they may have run in the matter, the surplus earnings should belong to the nurses alone, and can only be expended for their personal benefit. We feel sure that the correspondent from whom we quote cannot seriously suggest that any part of fees fairly earned by individual nurses should be diverted to the maintenance of a charity, however deserving.

### NURSES AT WOTTON-UNDER-EDGE.

Three years ago the District Nursing Association began work at Wotton-under-Edge with one nurse only, but it soon became apparent that there would be sufficient employment for a larger staff. A trained matron was therefore appointed, with three nurses under her, and the home was formally opened a year later. Since then a larger house has become necessary and private patients are admitted. The present staff consists of matron, twenty nurses, and one qualified midwife. Eight of these nurses are fully trained, but the others are in course of training for cottage nurses for the agricultural class. The cottagers are able to have a resident nurse for 2s. 6d. weekly, or any number of visits at a penny per visit, and a night nurse for sixpence a night.

### A DUBLIN TRAINING SCHOOL.

The introduction of a trained nurse as superintendent of the nursing department at the Mater Misericordia Hospital, Dublin, has justified the confidence of those who foresaw the advantages likely to follow the arrangement. The sisterhood finds that the probationers trained by Miss McGivney, the lady superintendent, do much credit to their fine old hospital, and are in much demand at the conclusion of their course. Some join the private nursing staff, which is said to be working most satisfactorily, and others follow useful careers in Union hospitals in various parts of the country. Besides the practical instruction which they receive in the wards, the probationers have lectures from the medical staff, who have shown much interest in the new departure of the nuns at the Mater Hospital in employing duly trained night and day nurses.

### NUNS AS NURSES.

Irish Guardians seem still much agitated on the "Nuns *versus* Nurses" question. At the Mallow Union it was publicly asserted the other day, as reported by the local Press, that "it was a well-known fact that the nuns never got, or needed, these paper certificates." We and many others have frequently pointed out the necessity for a nun to be systematically and properly trained if she is to follow the calling of a nurse. The Guardians themselves appear to understand that the qualities which they admire in the good women are the results of their preliminary training in convents, but, excellent as such discipline may possibly prove, it must still be supplemented by a full course of instruction in the theory and practice of modern nursing. No attack on the nuns has been made by the Local Government Board, although some of the Guardians wish to believe so; it is only when the latter place these good and pious women in positions for which they are not trained that the responsible authority protests at their action.

### QUEEN'S NURSES IN SCOTLAND.

The annual report of the Scottish branch of the Queen's Jubilee Institute, of which Princess Louise is president, was laid before a meeting of subscribers last month in Edinburgh. The chair was taken by Sir Douglas Maclagan, M.D., who moved the adoption of the report, which was agreed to. The Scottish council is responsible for some 30 nurses, and 58 branches have been affiliated with the Institute. In Edinburgh, during the year 56,307 visits have been paid, and 2,605 new cases cared for. The expenditure of the Scottish branch is reported to have exceeded the total receipts by £191 9s. 1½d., therefore it is desirable that immediate increased support should be forthcoming for the Queen's Jubilee Institute.

### NURSING IN RUSSIA.

The introduction of female nurses into Russian hospitals appears to date from the Crimean war, when the sick were found to be terribly neglected. The Emperor was unwilling even then to countenance the presence of women in the field hospitals, but he was eventually persuaded by his sister to consent to their admission. The Princess had many obstacles to face in carrying out her humane plan, for women nurses were previously little known even in civil hospitals; but her good example was followed by other ladies, and the employment of female nurses became in time established in Russia.

### SISTERS IN HONG-KONG.

Many kindly New Year's wishes have reached us from Hong-Kong, where the Government Civil Hospital is nursed by English-trained ladies. They write of great scarcity of water, owing to the small amount of rain which has fallen during the past year in the colony. The beautifully-kept hospital is delightfully situated, and our English sisters seem well content with their work and surroundings. The decorations at Christmas are confined to the European wards and general staircase, where they doubtless often bring pleasant memories of home to many of the patients.

### HOME COMFORTS IN THE COLONIES.

Sisters Aimée Blennerhassett and Lucy Sleeman have opened a private home for phthisical invalids and convalescents at Middelburg, Cape Colony. The house, situated in the outskirts of the village, is some 4,000 ft. above the sea-level, and the climate is a peculiarly dry one. An English doctor and clergyman reside in the village, and there is a public library, with reading and billiard rooms. Unlike most Karroo districts, Middelburg is well supplied with milk, butter, and vegetables all through the year. European servants and many home comforts are provided by the two English nurses for their patients.

### PRINCE ALFRED HOSPITAL, SYDNEY.

This beautiful hospital might well serve as a model for many English ones, and especially as regards the nurses' quarters, which are admirably planned and officered. Photographs of the buildings show that a profusion of shrubs and long stretches of lawn leave the back of the structure very open and airy, whilst a most extensive prospect of land and sea is obtained from the pretty front windows. "The nurses' home is a place to dream of," remarked an enthusiastic English probationer who recently travelled through Australia.

### DISTRICT NURSING IN MELBOURNE.

Considerable interest is shown in Melbourne with regard to the District Nursing Society there. The funds ought to benefit largely by the proceeds of the recent grand ball, which is the second given in aid of this deserving society. Lord and Lady Brassey were present at the entertainment, which was largely attended.

### A CHRISTMAS PROTEST.

We welcome with much pleasure the larger proportion of help which has been contributed this year by the general public towards Christmas festivities in hospitals and charitable institutions, especially in the provinces. All over the country comes evidence that willing hearts and hands, from outside as well as inside institution walls, have been busy making good cheer for the sick folk, and it is certain that their efforts have been much appreciated, not by the patients alone, but by the nurses, upon whose shoulders too much of this extra work often rests at Christmas time. And we take this opportunity of once again protesting against the "presentation" custom, which comes much to the fore at this season, and too frequently means a species of blackmail, nurses feeling compelled to join in giving presents far beyond their small means, for fear of possible misconception should their names be absent from the list of donors. No doubt many of these gifts are spontaneous expressions of a kindly feeling, but even as such they form an unnecessary tax on slender purses, and we would strenuously urge all matrons to discourage the custom to their own honour, and to the benefit of nurses.

### OUR NEEDLEWORK COMPETITION.

Most hearty acknowledgments have been received from the hospitals to which our parcels of clothing were sent, and the kindly workers will be glad to know how welcome their offerings have been. "Our sick have much valued the kind thought for them," wrote one matron, and another said, "The clothing is most welcome, and very pretty"; "The garments are beautifully made, and will be most welcome," wrote another recipient; and one and all wished their thanks conveyed to all those who contributed to The Hospital competition and distribution.

### SHORT ITEMS.

It has been decided by the guardians of St. George's, Hanover Square, that the senior nurses in their infirmary shall be called Sisters in future.—Nurse Ramsden has been appointed by the Treville Street District Nursing Society to visit the sick poor of Plymouth in their homes.—A Nursing Association has been formed in Urmston and a committee appointed to raised the necessary funds for the maintenance of a trained district nurse—It is proposed to secure a large house near the York County Hospital and fit it up as a home for the nursing staff.

### Lectures on Nursing.

By a Superintendent of Nurses.

### III.—DUST, DUSTING, AND DISINFECTION.

### Dusting.

One of the first things you have to do on entering the ward is to dust. This you may think has very little to do with nursing, but I want you to learn that it has a good deal to do with it. If you ever go into a room where the window shutters are closed, you will probably find there is some chink through which the sun is penetrating, and in that ray of sunlight you will distinctly see particles of various substances. Generally these motes or particles of atmospheric dust are invisible, and it is only when this strong sunlight is upon them that they can be seen, but whether seen or not they are always present in the air we breathe. These particles were formerly supposed to be inorganic matter like sand, but it has been proved by Professor Tyndall that air passed over a flame or through a red hot tube is entirely deprived of its particles, thereby proving that they were organic. Now I want you to realize this fact, that every patient in your ward is helping to pollute the atmosphere by filling it with harmful germs of disease. There are flying about shreds of linen from sheets, of wool from blankets, pieces of hair, epithelial scales, dried particles of pus and blood, crystals from urine, poisons from sweat, &c. You will thus see that dusting is a very important thing, and should be done with the utmost care, in a hospital especially. Not only should the tops of furniture be wiped, but every place where dust may lodge, skirting boards, ledges, bedstead rails, shelves, chair legs, table legs, &c. You should learn, too, the necessity of taking pains in dusting to avoid sending the dust from one place to another. A damp cloth should be used where it is possible, and if the duster is shaken out at all it should be outside in the fresh air, or over the fire, so that the dust may be carried up the chimney. Another piece of work which will fall to your lot will be the tidying of cupboards. As Miss Nightingale says, "These ought not to be receptacles of half-empty medicine bottles, stale food, and a miscellaneous mass," but should be in such order, that at any moment the doors may be thrown open and the cupboard inspected without your feeling uneasy. After dusting put your duster away in its place, do not leave this for someone else to do.

### Disinfecting.

The utmost cleanliness is necessary of all utensils used in a hospital, not a speck should be seen on spittoon, bottle, slipper, or bed-pan, and a little disinfecting fluid should be put into these before use. All utensils should be scalded after being used. If you have lived in the country and known anything of a dairy you will have learnt that the tins which have contained milk have always to be scalded when washed. If this is not done the milk soon turns sour. Take a saucepan, and boil some milk which is just turning, it will probably curdle, and you throw it away. Wash the pan with warm water till it looks quite clean, then put some new milk in it to boil. To your astonishment that, too, curdles. Yes! because your saucepan was not scalded.

Warmth encourages life, but intense heat destroys it; hence water should be at boiling point if it is to destroy germs. When milk turns sour, putrefaction has begun. What is meant by "putrid"? Some solids and fluids are more quickly turned putrid than others. If you take some blood and expose it to air, heat, and damp, the various parts of which it is composed will separate and give rise to foul gases, which are poisonous to a certain extent and more or less offensive to the smell. As these component parts separate, small organisms begin to appear. These organisms are the tiniest of tiny living things. Some idea may be formed of how small they are by the fact that one drop of impure water may hold almost as many organisms as there are men, women, and children all over the earth. These bodies are always to be found in stagnant water or in decaying animal or vegetable matter. They destroy the body after death. Directly life ceases bacteria appear; their germs or seeds are always floating about in the air. There are wholesome germs and poisonous germs. The air over drains is full of these last. They are waiting for a suitable place in which to develop. They lodge in the wrinkles of the skin; but while we are alive and the place on which they settle is clean and healthy these germs cannot enter the part and survive, while dirt or the death of the part, as in sloughing wounds, enables them quickly to flourish.

The bad gases before spoken of and the small germs of these bacteria escape into the air in favourable circumstances and float about, and when they enter any fluid such as blood or any solid that is liable to putrefaction they soon mature, and as bacteria cause this putrefaction to take place. In this way disease spreads. The decaying matter in which these bacteria always exist must be disinfected—that is, their life must be destroyed, and with their destruction putrefaction ceases.

"What, then, will destroy these germs?" you will say. Something is needed which will not only kill them when they are alive, but will also prevent them when lodged in an unhealthy part from coming to maturity at all; and it is the object of disinfectants to destroy or make harmless germs which under favourable conditions will prove most harmful. Antiseptic substances are those which prevent the growth of these small organisms, and which thus check putrefaction.

Germs of disease, such as small-pox and scarlet fever, escape into the air and spread the disease, and if no means are taken to destroy them they will lodge in human beings, who are then said to "catch" the complaint. From this you will see the very great need there is to disinfect all bedding used by the patient suffering from any infectious or contagious complaint, and not only the bedding and linen worn by the individual, but the air itself and all the furniture of the room in which the patient has been living. In order to disinfect the room one ought to have every aperture closed, chimney stopped up, and then sulphur put into an earthenware jar or plate and be set fire to, care being taken to place the jar or plate on an old tray so that the floor cannot by any chance be set on fire. If the walls are painted it is better to damp them first, in order that the fumes may in the moisture find a resting-place, as sulphurous acid is a powerful antiseptic in itself. Then when the fumes have been allowed to fill the room for twelve hours the doors and windows can be thrown open, and carpets, curtains (if any), and bedding should be sent to be steamed. A temperature of 250 deg. F. can generally be borne by such things. Then the walls of the room should be washed over with a weak solution of carbolic acid and the floors scrubbed with the same, skirting-boards, bedstead, chairs, &c., all being treated in like manner. As sunlight and oxygen kill many bacteria, a current of air should be constantly moving. All infected or soiled linen should be at once put into a pail and carbolic acid (1 in 20) poured on to it. It can be wrung out a little, and sent to the wash wet. A nurse should never let infected linen be carried through her wards in a loose, disorderly manner. It is best to bring the pail, if possible, to the bedside of any patient suffering from an infectious disorder. At all events, the greatest care is needed not to distribute the germs into the air by shaking the sheets about.

The poisonous germs of different infectious diseases are thrown off in various ways; for instance, the infection in small-pox is contained in the discharge from the pustules, that of typhoid fever in the evacuations from the bowels, that of scarlet fever in the secretions of the skin and mucous membrane, and in the peeling skin, that of diphtheria in the discharge from throat or nostril, and it is generally supposed that if these vehicles of disease become chemically altered by heat, oxygen, or disinfectants, the change would also affect the poisonous elements themselves. All secretions, therefore, as they are voided, and all matters expectorated or vomited, ought to be received into vessels charged with a sufficiency of disinfecting solution to cause chemical changes in the whole mass, thus effectually destroying the poison.

Vomited matter should always be inspected by the Sister unless she tells you it is unnecessary, as the doctor will rely upon her acquainting him with facts which will be of any assistance to him. A very large quantity vomited at one time would point to the pylorus being blocked. The pylorus is the opening from the stomach into the duodenum. If the vomit is very frothy he will gather that food ferments in the stomach. If blood is thrown up of a dark colour it may point to ulcer of the stomach or gastric congestion in heart, or liver disease. Then when there is obstruction of the bowels, as in strangulated hernia, fæcal matter is vomited. Sometimes when blood is thrown up it is changed in colour from red to dark brown, this being caused by the gastric juices acting upon it. Vomiting blood is called "hæmatemesis."

It is a good plan to have a piece of glass to cover any offensive matter so that it can be clearly seen without inhaling an unpleasant odour, and all such matter should at once be removed from the ward or room and kept in the lavatory.

### Food, with Special Relation to the Sick.

### II.—VARIETY IN DIET.

The second point we have to consider is that invalid food need not be monotonous. It often happens in a private house that if the cook has prepared a stock of beef jelly or chicken broth, there is a tendency to give the patient one or other of these without variety. This is partly due to a feeling that what is made must be used, partly because it is troublesome to plan a change of diet. No wonder, then, that a patient's frequent complaint is, "I am sick of eggs; I hate milk, and loathe beef-tea!" And unfortunately the patient is allowed to get into this frame of mind before anyone awakes to the necessity for more variety of food. But how much better for both had this dislike never been allowed to spring up.

To insure a proper change, we recommend the drawing up of a menu for each day in the week. This can be submitted to the doctor, if he does not, as is commonly the case in these days of highly-trained nurses, merely lay down the general principles of the diet he wishes given and leave the details chiefly to the nurse. If a nurse will draw up such a diet-sheet she will be surprised how much variety can be obtained even for a patient ordered to be fed every four hours on strictly fluid diet. Of course, when easily digested semi-solid food, such as light puddings, &c., is allowed, the menu can be considerably enlarged and more easily drawn up. The foundation of all invalid food must, of course, be nutritious, but there is no reason whatever why milk and beef-tea should be the sole articles of diet. Instead of serving up this monotonous beef-tea, time after time, a good soup should be given, which can be quite equal to beef-tea, and made of fresh meat but flavoured by boiling in it some fresh vegetables and herbs, which are to be removed afterwards by straining if no solid food is permitted. The nutrition in the soup is by no means lessened by the addition of the salts which abound in vegetables, whilst its flavour is immensely improved.

Again, milk need not always be given in its simplest form, but can be made into white soups with a variety of flavourings. Many a patient would readily take half-a-pint or more of milk thus disguised, or varied by the addition of a little Valentin's Beef-juice, or some Liquor Carnis, or other approved meat extract, with vegetables and herbs as already described.

An excellent soup can be made of milk and the white meat from the breast of a fowl pounded up in a mortar into a thorough paste.

Then also milk and meat juices can each be made into jellies with the simple addition of gelatine and suitable flavourings, or Sir Henry Thompson's "Invalid's Aspic Jelly" would make a pleasant change. It has been the custom to attach no importance to jelly as an invalid's food, but so great an authority as Dr. Burney Yeo has lately shown that, although possessing no nourishment in itself, a certain amount of jelly is useful in preventing too rapid tissue waste in fevers. So far it has been assumed that the patient can digest milk, but unfortunately this is not always the case; and then it is often very difficult indeed to vary a milk diet. The indigestibility of milk lies in the curd which is formed in the stomach, and where the curd is firm and hard, like that of cow's milk, it is very difficult to digest. Hence the use of whey, from which the curd is removed, which, when sweetened with a little milk sugar, forms a very pleasant though not very nutritious drink. Some invalids can take milk when the curd has been artificially produced and pounded up, and returned to the whey. Also the addition of lime or soda water to milk prevents so hard and firm a curd from forming. Sometimes fermented milk, known as "Koumiss," agrees with an invalid.

The advisability of allowing fever patients tea or coffee is still a moot point. Some doctors approve of it whilst others do not. When permitted there is no doubt that it is much appreciated by the patient. It must be remembered in connection with these beverages that there is no nourishment in tea or coffee in themselves but only in the milk. The tea must always be freshly made, and enough tea used to get the proper strength without allowing it to "draw" too long. The best kind of tea to use is that from China, in preference to that from Ceylon or India, as the latter kinds contain more tannin, and part with it more readily even than the Chinese. Unfortunately tannin is so exceedingly soluble that it is impossible to have tea without a trace of it. Some authorities say that a pinch of bicarbonate of soda counteracts the tannin, and at any rate it has the advantage of being both a simple and a harmless expedient.

Milk and cream should be added to tea, and also a well beaten-up egg, and thus it can be made a nutritious drink in moderation, care being taken not to add the tea too hot, or the egg will curdle and lie on the top.

Coffee can be made far more nutritious than tea, and has the advantage of containing no tannin. A small quantity of very strong coffee added to a cup of hot milk, and, if advisable, a beaten-up egg, contains the maximum of nourishment. Moreover, both tea and coffee can be infused with boiling milk instead of water.

Cocoa, also, made entirely with milk is excellent, and this or coffee, or each on alternate mornings, forms an excellent breakfast for a sick person, and preferable to plain milk, whilst a cup of tea, made as already described, can be given in the afternoon about four o'clock.

In addition to what may be called regular meals, a fever patient requires beverages simply to allay thirst, and it may, or may not, be advisable that these should also be nutritious. Amongst the latter are rice or barley water, milk gruel, milk with soda water, &c.; whilst amongst the non-nutritious may be mentioned imperial drink, lemonade, and various fresh fruit juices, or syrups, to which plain water or soda water is added.

In sick diets we must be guided by the same rules that regulate diet in health, and whilst consulting the idiosyncrasies of our patient, must see that the food supplies the body with materials to renew all the tissues. We must also take into account the time required for the digestion of different foods, and select those which are digested quickly, especially in cases of great weakness.

### Practical Aspects of a Nurse's Life.

By Sister Grace.

### I.—AN ADMONITORY INTRODUCTION.

Many and varied are the causes which lead to the choice of nursing as an occupation by the shoals of young women who apply for admission as probationers to our London and provincial hospitals. Very many—the largest proportion probably of those who become nurses—do so because it is necessary that they should earn their own living in some way, and they think that nursing will be an easier method, and, as they would express it, a more "genteel" way than as servants or dressmakers. I need hardly say to those who know anything of the subject that the life of a general servant is in most respects much easier than that of a hospital nurse.

### Nurses In and Out of Novels.

Next comes a large and inreasing section of society where young ladies have a great deal of time on their hands; and, as is frequently the case at the present day, they are dissatisfied with their lot in life, find it dull, narrow, and uninteresting, and think they discern a picturesque opening as hospital nurses. They have read novels—generally great rubbish—where the nurses are always charming creatures, attired in becoming costumes, who glide from bed to bed, smoothing pillows, moistening hot brows with eau de cologne, and performing other light and elegant duties which seem to be the entire occupation of nurses in novels. The end of these charming creatures is always matrimony, their only difficulty being to choose among the host of enamoured house surgeons who hang upon their smiles. There is nothing said in these novels of rising in the dark, sweeping dusty wards, making the beds of 20 ro 30 patients in a more or less disagreeable atmosphere, making up fires, polishing tins, and scrubbing sculleries till the hands become coarse, rough, and black; nor of the very great difficulty the charming creature experiences in keeping the becoming costume relatively clean. Nothing is said of the difficulty of pleasing sisters and staff nurses, or of overcoming the querulous prejudices of patients by no means invariably charmed with the new "pro"; nothing of the aching back and feet, nor of the heart sickness that comes over one, when day after day goes by in much the same routine of hard uninteresting work, for it is necessarily some time before the new and useless "pro" emerges from the scrubbing and cleaning stage to see a little more of nursing.

A probationer must always learn to scrub and clean if she is to be worth anything; but after the first few months, if she is a capable and intelligent person, she will be allowed to help other more experienced nurses with the dressings and general management of the sick.

### "Born" Nurses.

Lastly comes the smallest and most valuable section of candidates, those who take up the work, not because they must earn their living at something, nor because they are dull and dissatisfied at home, but because they feel an irresistible attraction to the work. One often hears of a "born" nurse, and there is something in the term. Being a "born" nurse will not enable one to do without a strict and severe training, but on the other hand, I think no amount of training will make a really satisfactory nurse without having in oneself that natural aptitude for nursing and tending the sick—that mechanical quickness and lightness of touch and that untiring sympathy and patience which contribute to the quality known among the lower classes as being a "born" nurse.

### Private Nurses.

By far the larger number of women who enter our hospitals for training eventually become private nurses; that is to say, nurses who, after their hospital training of two or three years is over, become attached to the staff of some institution, and from there are sent out to nurse the sick, poor and rich alike, at their own houses. For this class of nursing a somewhat long experience leads me to think that superior upper servants, parlour maids, ladies' maids, and housekeepers, &c. . . . make the best nurses. They have learnt by long practice nice little ways of waiting upon people, and when a nurse has learnt that she has acquired one very important item in her nursing experience.

### Sisters of Wards

Should, I think, always be chosen from the upper classes for many reasons. Ladies, as a rule, have a wider knowledge of the world; and the broader our views of life the more lenient we are inclined to be to the errors and shortcomings of those with whom we associate. Then, as a rule, ladies have more self-control and tact than those who spring from the classes below them, and also more courtesy of manner. All these qualifications are greatly needed in one who has control over patients, nurses, servants, and porters. Moreover, there is on the part of nurses generally a not unnatural prejudice in favour of having over them one who is a lady by birth, breeding, and disposition.

### Staff and Night Nurses.

It is certainly desirable that both these types of nurses should be women of tolerable education and refinement. Their work also requires a certain amount of tact and considerable judgment. They, too, have to exercise control over probationers and others who work under them. The position of a staff nurse is by no means always an enviable one; she has to issue orders and control other nurses without having the full authority of a sister; she is a species of middleman, if I may so use the term, and sometimes finds it difficult to exert due control over those under her without trenching upon the prerogative of the sister. Probationers should try to remember that the position of a staff nurse is a difficult one, and by cheerful and willing obedience try to make her life as easy as possible. A night nurse in the wards needs to be a woman of exceptional conscientiousness and loyalty. She is working to a very great extent without supervision, and has many opportunities, if so inclined, of shirking a great many minor duties. In my experience of eight years as a sister I can speak most gratefully of the excellent and conscientious work of my night nurses. As a rule they did their work "not with eye service as men pleasers, but as the servants of Christ, doing the will of God from the heart."

### Hints to Beginners.

Now let me give a piece of advice to anyone who is thinking of entering the nursing profession. Count the cost first, and then you will not be disappointed. Remember that if you are to be a really good and valuable nurse you must not allow anything else to absorb even a portion of your life. Nursing is an exacting occupation, and to do it well everything else must be given up—society, friends, even home ties. Nothing must enter into competition with your nursing life. It is a grand and noble life, but unless you can give up all to carry out that life, so that having once put your hand to the plough you will not wish to look back, do not enter upon it. Perhaps you think this is a hard saying of mine, but I speak from experience. I advise all to think the matter out thoroughly before embarking on a life which, happy as it is, means a life of entire self-sacrifice. On account of the difficulty of keeping up home ties, I think women who are only daughters or only sisters are not the most suitable to become nurses. The dissatisfied nurses I have seen were those who tried to keep up two lives, one in nursing and one in the world. I think the great happiness I have experienced in my eleven years of hospital life is greatly due to the fact that I resolutely put all else out of my life, and so I had no uneasy longings after the gaieties and social pleasures of my past life.

### Christmas Entertainments.

Christmas Day began with carols sung by the nurses at the London Hospital, and ended with excellent entertainments in all the wards. No pains were spared by either medical or nursing staff to ensure a happy day to the patients, and their efforts were crowned with unqualified success. On the 30th ult. the Christmas tree entertainment took place in the children's wards, and a large number of guests afterwards inspected the hospital and the nurses' home.

The decorations at St. George's Hospital were very elaborate, and the wards looked quite gay. All the patients received useful gifts, and the children had a goodly share of toys.

The pretty wards at the London Temperance Hospital looked very bright with electric light and plants and flowers when they were inspected by many visitors on Monday evening. The entertainment for the patients and guests took place in an unoccupied ward, which was gracefully decorated for the occasion. For the nurses' entertainment an equally attractive programme was arranged for January 3rd.

A large number of guests were present at University College Hospital on the 30th ult., when an excellent entertainment was provided for the patients. A pretty Christmas tree was illuminated, and the distribution of the gifts from it to the inmates of the hospital was a much-appreciated feature of the evening.

Christmas was celebrated in a pleasant fashion at the Great Northern Hospital, where the patients and nursing staff spent a very agreeable day. Gifts were provided for all the patients, and the hospital looked exceedingly bright and festive.

As usual, the festivities at Guy's Hospital occupied Christmas week, and only ended with the Old Year. The musical and other entertainments were thoroughly enjoyed by the patients.

Every patient at St. Bartholomew's Hospital received a useful gift on Christmas Day, and in the afternoon visitors were admitted to the wards. In Sitwell and Lucas Ward there were famous Christmas trees, whilst a gigantic wedding cake caused great amusement in Hope Ward, especially when it was found to contain prize packets for the patients. The evening concluded with entertainments in the several wards.

The wards at Westminster Hospital were prettily decorated, and suitable gifts were presented to each patient. An excellent dinner was provided and carols were sung by the staff, bran pies and other amusements combining to make Christmas very enjoyable to the patients.

The patients at St. Thomas's Hospital were regaled with seasonable fare, and each of them was permitted to invite one friend to tea. This privilege was highly appreciated, and the meals so daintily spread in the several wards proved amply sufficient for the assembled guests. The liberality of the medical staff at St. Thomas's provides for both ward decorations and this extra hospitality. The Christmas tree entertainment for the children passed off most successfully.

At Charing Cross Hospital the wards were decorated very tastefully indeed, and the tea tables were as pretty as usual. At six o'clock a fine tree loaded with gifts was lit up in the board-room, and all patients who were able to be down there enjoyed the carols sung by the nurses whilst they awaited the distribution of their presents.

A christmas tree in Moore Ward was one of the chief attractions to the children at the Royal Free Hospital, and the adult patients also received seasonable gifts. Visitors in the afternoon and entertainments in the evening made the wards very busy all day long, and the patients were provided with plenty of suitable fare for the festival.

On Christmas Day the patients of the Middlesex Hospital were regaled with turkey, plum-pudding, &c., and received a large number of gifts previously collected by the lady superintendent. The wards, prettily decorated with palms, ferns, and flowers, were illuminated in the evening with fairy lights and coloured candles. For tea a special provision of cake was made by Messrs. Buszard. A monster Christmas tree, erected in the board-room, was unladen of its treasures in the presence of all the patients able to leave their wards, while those confined to bed were visited by a large bran-pie containing their presents. From a smaller tree in the children's wards gifts were distributed on New Year's Day.

At St. Mary's Hospital, Christmas fare was provided, and various entertainments, concerts, &c., have made the festive season very pleasant to the patients. The Christmas tree entertainment was arranged for January 3rd.

The inmates of the Hospital for Epilepsy and Paralysis, Regent's Park, had a very happy Christmas, there being only three vacant beds. A fine doll and other toys, sent by the Editor of *Truth*, were appreciated by the younger patients, and all the inmates enjoyed games, songs, and substantial fare, the dessert being provided by the paying patients and their friends. The matron, Miss Miriam Ridley, was presented with a beautiful dessert service by *the members of the nursing staff*.

Varied and plenteous fare was provided for the inmates of Caterham Asylum on Christmas Day, and mirth and contentment reigned throughout the vast institution. The wards were artistically decorated, the mental nurses showing themselves to be in no way behind their hospital sisters in refined taste. A handsome dinner was provided for the nurses after the patients had finished their feast.

The addition recently made to the East End Mothers' Home of a large room in the rear of the original building enabled Miss Blomfield to give a pleasant little entertainment on Boxing Day to her patients and their husbands. The pretty tea-table was furnished by private friends, and the gigantic bran-pie was also stocked by their kindly aid. Oranges, buns, and bags of sweets were carried away by "the fathers" for each of the children at home, and the possession of these gifts for their little ones appeared to add considerably to the parents' enjoyment of their own party. All present received some useful garment for themselves or "the baby," and the evening concluded with a vote of thanks, cordially proposed by one of the fathers, to their kind hostess. Three cheers for "The Babies" followed, and those who had helped to entertain were not forgotten, "three cheers for the lady who told us the stories" being accompanied by a low-voiced comment from a working man, "And I know as this is the first Christmas as I've ever rightly enjoyed in my life!"

The wards at Chelsea Infirmary were decorated with excellent taste, each having a distinctive colour or combination of shades. The patients had their usual Christmas dinner, and did ample justice to the roast beef and plum-pudding. The nursing staff had turkeys, geese, plum-pudding, mince-pies, and dessert on the following day. The children's wards attracted much admiration, both on account of the charming decorations and also the well-cared-for aspect of the little patients, who were picturesque in red flannel dressing-gowns; or, in the case of the convalescents, in red frocks and white pinafores. The Christmas tree was a centre of attraction for all the little folks.

At the Hospital for Consumption at Brompton a gorgeous Christmas tree entertainment was held on New Year's Eve, and other seasonable pleasures were provided for the patients last week. The entertainment, as usual, was largely attended and highly appreciated.

### Christmas in the Provinces.

HUDDERSFIELD INFIRMARY. — Christmas fare marked Christmas Day, and on December 30th the annual Christmas tree and entertainment provided by the Ladies' Committee was held. Each patient received some useful article of clothing, and every nurse and servant had a present, most of the nurses choosing a text-book on nursing or cognate subject. Recitations, songs, and conjuring followed, and were much enjoyed by the patients and numerous friends of the institution, who had assembled in the large accident ward, which, like the others, had been prettily decorated. On the 31st ult. the supper and dance for nurses, servants, and residents took place. The board-room was set apart for light refreshments, and, after the supper provided by the Ladies' Committee, an adjournment was made to the out-patient room, which, in its pretty drapings of art muslin, made an excellent ball-room, where all present thoroughly enjoyed dancing the old year out and the new one in.

LINCOLN COUNTY HOSPITAL.—Christmas this year was started by a concert on the 18th ult. given by the Hospital Saturday Committee. On Christmas Day the patients partook of a sumptuous dinner of pheasants, roast beef and plum-pudding. A tea was held in the Johnson (female) Ward, followed by music and games. Every inmate received some useful present and Christmas letter by their bedsides. The convalescent male patients enjoyed some tobacco in the out-patient room, and were entertained by the assistant house-surgeon. The matron and nursing staff—26 in number—dined at half-past six in the evening. On the 27th ult. two performances of Punch and Judy were given in the children's ward, also a tea party in the Dixon Ward. On the 30th ult. a magic lantern was shown in the children's ward, and was much appreciated by everybody able to be present. A New Year's Eve tea party was given in the children's ward. Afterwards Sister Ruston distributed from a well-laden tree the various articles to the delighted little ones. The ward decorations were unusually light and pretty, coloured crêpe paper relieving the heaviness of the evergreens. Matron, sisters, and nurses are to be congratulated for the excellent manner which they arranged everything for the enjoyment of the poor suffering inmates.

CHRISTMAS AT LINACRE FEVER HOSPITAL.—The convalescent ward at this hospital presented a most gay and festive appearance. The chief feature of all the decorations was the Christmas tree, laden with toys and useful presents. The patients, and especially the children, were delighted, and entered into the spirit of the entertainment with so much zest and evident enjoyment that the nurses felt doubly repaid for all their efforts to make Christmas Day a happy time for them. Unlike those in general hospitals who look forward to seeing their friends, these poor patients, owing to the nature of their disease, are deprived of this pleasure, as they are also cut off from other amusements, such as concerts, &c., so frequently given by charitable ladies and gentlemen in other hospitals. It is only right that Christmas Day in fever hospitals should be made doubly happy for the patients, if only to dispel the sense of isolation they must feel at this time especially. The Christmas tree was a thoughtful gift from a gentleman who evidently reasoned that fever patients deserved as much sympathy from the outside world as the patients in non-infectious hospitals. The toys and useful articles which adorned the tree were the result of an "appeal" inserted through the kindness of the editor in the local paper. Needless to say, it was responded to in a most generous way, showing that if these matters were brought forward a little more clearly before the public their sympathies would be quite as much in favour of providing the means for Christmas festivities in fever hospitals as in the more favoured general ones.

WHITCHURCH COTTAGE HOSPITAL.—At the invitation of the matron a number of the patients past and present met the committee and staff at a musical evening on Friday last, 27th ult. The programme was supplied by many ladies and gentlemen of the district, to whom a hearty vote of thanks was accorded on the motion of the Chairman, seconded by the Hon. Secretary. At the interval refreshments were provided for the patients and visitors, the cost of which was defrayed by special donations.

NATIONAL CHILDREN'S HOSPITAL, DUBLIN.—The National Children's Hospital in Dublin is not a very large institution; not nearly so spacious as those interested in its welfare would like it to be, numbers of applications for admission being rejected every year from lack of funds to support an increased number of patients. The daily average number of beds occupied in the hospital is thirty-three, and the little patients are well cared for, as their bright faces amply prove. "The League of Kindness" gives Father Christmas a large share of invaluable help in the amusement of the children, the supply of toys and new sixpences, which has never failed at this season since the League was instituted, being by no means the least of his pleasant surprises. Minor entertainments also had their place in the Christmas programme, and those who know how children can enjoy themselves will not need to be told how very thoroughly the little patients appreciated every treat, or how well everyone connected with the hospital felt rewarded for the extra trouble which Christmas brings by the sight of their bright faces and the sound of their merry laughter.

### Presentations.

THE nurses of the Kingston Nurses' Home, Hull, have presented Miss L. Wood, their lady superintendent, with a gold curb bracelet as a token of their esteem.

ON Christmas Day the nursing staff of the Norwich Isolation Hospital presented the matron, Miss Bayne, with a handsome crocodile photograph screen.

MRS. SMITH and Miss Duncan, lady superintendents of the Blackheath and Richmond Nursing Institution, were presented on Christmas Day by the nurses with a handsome silver salver.

THE nurses of the City Hospital for Infectious Diseases, Newcastle-on-Tyne, have presented their matron, Miss Ogston, with a brass gong and silver sweetmeat tray. Miss Ogston has been matron of this hospital for fifteen years.

### Notes and Queries.

The contents of the Editor's Letter-box have now reached such unwieldy proportions that it has become necessary to establish a hard and fast rule regarding Answers to Correspondents. In future, all questions requiring replies will continue to be answered in this column without any fee. If an answer is required by letter, a fee of half-a-crown must be enclosed with the note containing the enquiry. We are always pleased to help our numerous correspondents to the fullest extent, and we can trust them to sympathise in the overwhelming amount of writing which makes the new rules a necessity. Every communication must be accompanied by the writer's name and address, otherwise it will receive no attention.

**Queries.**

(66) *Lady Roberts' Nurses.*—Where can I obtain information about Lady Roberts' Nursing Institution?—*Sister Alice.*

(67) *Temperature Charts.*—Where can I obtain Maw's temperature charts?—*F. A.*

(68) *Neuheim Treatment.*—Can you tell me where I can obtain instruction in the new Neuheim treatment?—*Q. E. D.*

**Answers.**

(66) *Lady Roberts' Nurses.*—"THE HOSPITAL Nursing Supplement," p. cxxxvi., July 1st, 1893.

(67) *Temperature Charts.*—Write to Maw, Son, and Thompson, 7, Aldersgate Street. The Scientific Press, 428, Strand, W.C., also can supply you with very good temperature charts, price 1d. each, 1s. 3d. for 25, 2s. for 50.

(68) *Neuheim Treatment* (*Q. E. D.*).—Write to Dr. Bezley Thorne or Dr. Fletcher Little.

### French Schools for Trained Nurses: Their Origin and Organisation.

By Madame W. Vignal.

At the Asylum of St. Anne there are not so many diplomas awarded as at the Bicêtre and La Salpétrière, a fact to a great extent explained by the small number of pupils attending the classes organised at this asylum; under one hundred and fifty. In the male school there are seventy-four pupils, in the female sixty-eight, composed of male and female head and ordinary nurses; about fifteen outdoor pupils complete the number.

In 1888 ten diplomas were granted, in 1889 eighteen, in 1890 seventeen, in 1891 nine, in 1892 ten, in 1893 thirty-two, and in 1894 forty-six.

The increase in number in the last two years warrants the hope that the number of St. Anne pupils will steadily increase.

The general council, though refusing to grant the hospital subordinates the advantages accruing from the law passed on June 19th, 1853, in relation to the civil pension, have decreed that the subordinates of St. Anne's Asylum should receive annually an indemnity equal to a retiring pension. Nurses of all grades receive an indemnity equal to half of their salary. After twenty years' active service they receive a pension according to their grade, varying from £21 to £60 per annum. The salary given is quite inadequate to the services rendered, and insufficient as a livelihood, and the day is hopefully looked forward to when widows and children of the hospital subordinates will inherit the pension of the yearly distribution of purses instead of that of books.

Saving bank "livrets" from 18s. to 25s. are given to the indoor pupils, thus increasing their scanty pay; outdoor pupils only receive books.

The general council, in order to encourage the male and female nurses to obtain a diploma, has decided to grant to those who distinguish themselves an annual increase of salary.

At St. Anne's Asylum adult lunatics only of both sexes are received, of whom about a thousand can be lodged; children are sent to Bicêtre and elsewhere.

The nursing staff are all dressed nearly alike, without distinction of rank; their clothes are made of thick cloth, which is worn both in winter and summer. The food provided for the nursing staff in this asylum is by no means monotonous; on the contrary, it is frequently varied. The early breakfast consists of milk; the lunch, or second breakfast, comprises two courses—one of meat, another of vegetable, and dessert. The dinner consists of soup, meat, vegetables, and dessert.

This school provides families with male and female nurses, and affords the same convenience to other asylums, thus creating a career for those who wish to become properly qualified nurses.

### Appointments.

Branch Seamen's Hospital.—Miss Calvert, matron of the Newark-on-Trent Hospital, has been appointed Matron of the Branch Seamen's Hospital, Royal Victoria and Albert Docks. Miss Calvert was trained at Guy's Hospital, and was subsequently sister at the East Dulwich Infirmary. For the last six years she has been matron of the Newark-on-Trent Hospital, and takes with her many cordial good wishes. We wish her continued success.

### The Princess Maud Marriage Present Fund.

The list of contributions to the marriage present from the Nurses of the Royal National Pension Fund to the Princess Maud has now closed, and we are glad to announce that the amount received is (£53) fifty-three pounds, so that upwards of one thousand nurses have sent in contributions. This sum will enable a very suitable gift to be selected. We have received a good many suggestions from nurses as to the form the present shall take, and after the meeting of the nurse representatives on the Council the final decision will be announced.

Amount previously acknowledged ... £51 9s. 0d.

Seventh and Final List.

| | s. | d. | | s. | d. |
|---|---|---|---|---|---|
| L. B. Mobbs ... ... | 1 | 0 | S. A. G. ... ... ... | 2 | 6 |
| Nurse Howard ... ... | 1 | 0 | M. Horton ... ... ... | 1 | 0 |
| Nurse Lees ... ... | 1 | 0 | K. H. ... ... ... ... | 1 | 0 |
| Nurse Garratt ... ... | 1 | 0 | Policy No. 3,311 ... | 1 | 0 |
| Nurse Meyer ... ... | 1 | 0 | K. Lundley ... ... | 1 | 0 |
| A. Conway... ... ... | 1 | 0 | Policy No. 2,900 ... | 1 | 0 |
| A. M. Quantrill... ... | 1 | 0 | Policy No. 2,170 ... | 1 | 0 |
| Policy No. 72 ... ... | 1 | 0 | G. B. Elloogan ... ... | 1 | 0 |
| Policy No. 1,142 ... | 2 | 0 | J. C. Kercher ... ... | 1 | 0 |
| E. J. Edwards ... ... | 1 | 0 | A. R. Bellamy ... ... | 1 | 0 |
| K. Pitts ... ... ... | 1 | 0 | M. Chinn ... ... ... | 1 | 0 |
| A. M. Coppin ... ... | 1 | 0 | G. L. Travis ... ... | 1 | 0 |
| Policy No. 3,785 ... | 1 | 0 | B. C. H. ... ... ... | 1 | 0 |
| W. Pauline... ... ... | 1 | 0 | | | |

Received per Royal National Pension Fund.

S. Williams ... ... ... 1s. 6d.

Grand Total ... ... ... ... £53 0 0

### Novelties for Nurses.

### POCKET DISINFECTORS.

Disinfection is now a subject of general interest, and it is a matter of satisfaction in the household as well as in the institution when its theories are felt to be efficiently carried out. Before the variety of disinfectants now in use had sprung up individuals relied very much upon carrying the talisman about on their persons. This talisman usually took the form of camphor, secured in small bags or perforated boxes. Camphor has had its day, and nervous people and others can no longer find consolation in its possession about their person. Yet there are many who would be more at ease when running the risk of infection, in omnibus, railways, and insanitary quarters, were they to have a disinfecting agent close at hand. The "Sanitas" Company have realised the want, and their little pocket disinfectors, very like the cherished camphor bag of old, will be hailed with gratitude by many. We need not sing the praises of "Sanitas," which forms the basis of these disinfectors—its excellence is too well known—but we recommend to notice this new and convenient form of its application.

### AN EXCELLENT PRESERVE.

It becomes more difficult every year to procure preserves made from sound, wholesome fruit without the addition of some foreign substance. Messrs. Chivers, of Histon, Cambridge, whose name is already well known in connection with the useful form in which they have prepared table jellies, now supply the most excellent strawberry jam that it is possible to buy. The fruit is of good size and colour, clearly and temptingly preserved, and keeps admirably. It is not possible to give much higher praise than this, yet we are not afraid that our readers will deny the truth of the assertion if they essay the preserves for themselves.

For Everybody's Opinion, see cxviii.

Now Ready. Third and Revised Edition (twenty-fifth thousand), demy 16mo. (suitable for the apron pocket), handsomely bound in cloth boards, 160 pp., price 2s. In handsome leather, gilt, price 2s. 6d., net.

### THE NURSE'S DICTIONARY

### OF MEDICAL TERMS AND NURSING TREATMENT.

**Compiled for the use of Nurses, by HONNOR MORTEN.**

Containing descriptions of the Principal Medical and Nursing Terms and Abbreviations, Instruments, Drugs, Diseases, Accidents, Treatments, Physiological Names, Operations, Foods, Appliances, &c., &c., encountered in the Ward or Sick-room.

"Very useful for reference, and should be at the disposal of every nurse. It comprises not only what its name implies, but a description of the common abbreviations used, of many instruments, drugs, &c."—*Birmingham Medical Review.*

"A small and convenient manual which can be consulted at a moment's notice."—*Queen.*

---

*Just Published, 8th Edition. Greatly enlarged and thoroughly revised. Crown 8vo., cloth gilt, 3s. 6d.*

### NURSING: ITS THEORY AND PRACTICE,

BEING A

### COMPLETE TEXT-BOOK OF MEDICAL, SURGICAL, AND MONTHLY NURSING.

**By PERCY G. LEWIS, M.D., M.R.C.S., L.S.A., &c., &c.**

To this edition have been added three chapters on Monthly Nursing, together with additions to the chapters on Blood Diseases, on Antiseptics, on the Nursing of Children, and on Private Nursing. Under these headings will be found a short account of the new Aseptic Treatment, the Antitoxin and Serum Treatments, and the Treatment by Thyroid Extracts.

These additions, combined with the revision throughout of the text, render this work the best and most complete Text-book on Nursing in the market, and strengthen its position as a Classic in the Training Schools of the World.

"We have read the book with interest and can warmly recommend it as a nursing manual. . . . . Full of useful hints and information."—*Birmingham Medical Review.*

---

Just Published. With numerous Illustrations, demy 8vo., blue buckram, gilt, 3s. 6d.

### DIET IN SICKNESS AND IN HEALTH.

### By Mrs. ERNEST HART,

*Formerly Student of the Faculty of Medicine of Paris, and of the London School of Medicine for Women.*

**With an Introduction by Sir HENRY THOMPSON, F.R.C.S., M.B. Lond.**

SIR HENRY THOMPSON, in his Introduction, says: "I do not hesitate to express my opinion that the present volume forms a handbook to the subject, which will not only interest the dietetic student, but offer him, within its modest compass, a more complete epitome thereof than any work which has yet come under my notice."

"The importance of the subject to which Mrs. Ernest Hart has devoted this volume will not be disputed by any practical medical man. As to how Mrs. Ernest Hart has discharged her self-imposed task we prefer to quote from the introduction the words of Sir Henry Thompson, than whom there could be no more competent critic of a subject which he has himself so much illuminated by his admirable essays."—*British Medical Journal.*

"Medical men will find much of the information given very useful in daily practice. The chapters dealing with the principles which underlie scientific dietetics are characterised by common sense, and are replete with practical suggestions and directions. The chapter on the alimentation of diabetics is of special excellence. Not only are the general rules of diet in this morbid condition carefully discussed and explained, but we have a complete scheme of diet with *menus* for a whole week, showing what a vast amount can be done to palliate the irksomeness of the restrictions by a little ingenuity, forethought, and resource. On the whole we endorse Sir Henry Thompson's recommendation of the book as 'supplying an important want in our educational literature.'"—*The Medical Press.*

---

Third and Revised Edition, demy 8vo., 200 pp., profusely illustrated with Copyright Portraits and Drawings, price 2s. 6d.

### HOW TO BECOME A NURSE: AND HOW TO SUCCEED.

A complete guide to the nursing profession for those who wish to become Nurses, and a useful book of Reference for Nurses who have completed their training and seek employment. Compiled by HONNOR MORTEN (Author of "Sketches of Hospital Life," "The Nurse's Dictionary," &c.).

"A book that I can most cordially recommend to the notice of any woman or girl desiring to enter the ranks of this profession. The style is bright and interesting and the matter excellent. The volume contains all sorts of useful hints most happily conveyed."—*Woman.*

---

LONDON: THE SCIENTIFIC PRESS (LIMITED), 428, STRAND, W.C.

### Everybody's Opinion.

[Correspondence on all subjects is invited, but we cannot in any way be responsible for the opinions expressed by our correspondents. No communications can be entertained if the name and address of the correspondent is not given, or unless one side of the paper only be written on.]

### NURSES AND UNIFORM.

"Another Provincial Trained Nurse" writes: I am glad to see letters by a "Provincial Trained Nurse" and others in recent issues of The Hospital protesting against the terrible abuse of our dress. Other officials have their uniform respected, and why not nurses? A short time ago it was discussed in The Hospital whether it was best for nurses to wear or to discard outdoor uniform. Now there are a large proportion of women who are obliged to wear it, such as district nurses (of whom I am one); private nurses while engaged at cases, and many hospital nurses. But it is enough to shock and discourage any "trained" nurse to see the unblushing way in which uniform is copied by untidy and (often) fast-looking women—especially in the West-end of London—one sees them with dyed hair and dresses draggling through the mud. Nowadays a distinctive dress does not prove anyone to be a respectable character. Roughly speaking, three sets of people wear uniform, namely, trained nurses, domestic servants, and women of bad character. Can nothing be done to protect it?

### FOOD AND HOURS IN ASYLUMS.

"Ex-Nurse" writes: In a recent number of The Hospital suggestions are invited as to the best remedy for the complaints of asylum nurses and attendants. I will take the food question first. As a rule, the raw food is pretty good and in sufficient quantity. Nurses and attendants would fare well if it never went near the kitchen; if they cooked it themselves or a cook was employed for attendants and nurses alone. In large county asylums there is one paid cook, assisted by inexperienced girls, who constantly leave because they are overworked and badly treated. The cook's time is devoted to fancy dishes for the medical staff and petty officers, and the relations they may have visiting them. The attendants' food is, consequently, spoilt by girls, assisted by patients. Is it to be wondered at that the meat cooked quickly in a gas oven is burnt to a cinder outside and quite raw inside? Cabbage is often soaking in water, slugs, and caterpillars; potatoes soddened in steam and sent up full of water. Rice puddings are made with separated milk well diluted. We are favoured with a treat in the shape of Yorkshire pudding, and I should like to send the editor a sample of this dainty, but if given to the office boy he would never get home to see his mother again! It is not surprising that nurses live on surreptitiously-made tea and bread and butter; become anæmic without complaint, preferring to suffer rather than be accused of grumbling by junior officials, who behave as if the place belonged to them. In asylums where head attendants and head nurses take their food with the attendants and nurses there are few causes for complaints, and the reason is obvious. Why should there be distinction between the food, and why should officers be allowed stimulants in teetotal county asylums when they are denied to attendants? The question of hours is being discussed in various quarters, and ought to be shortly before Parliament. The late Factory Bill dealt with laundries or workshops where machinery is used, restricting the hours of labour for women, compelling employers to allow an hour for dinner. Somehow or other asylum laundries have been forgotten, the laundrymaids are compelled to work all hours like the kitchen girls, their little bit of "time off" often stopped for frivolous reasons. Attendants are at last airing their grievances, and now that certificates are required, and qualified nursing is taking the place of the Sarah Gamp school, it is only reasonable that proper treatment, better food, and shorter hours of labour should be granted.

### NIGHT AND DAY DUTY COMBINED.

"An Old Hospital Sister," of seventeen years' standing in the profession, writes: I beg permission to reply to "An Old Time Nurse" who writes on the subject of ovariotomy cases being nursed night and day by the same nurse, and in answer can only say that, looking on such a practice calmly and with an unprejudiced opinion, it seems to me io be a most decidedly unwise and risky way of nursing so very important an operation, knowing, as a nurse ought to know, of how much vital importance it is that the woman who undertakes the care of such a case be fresh and bright, and never for a moment during the early days following such a grave operation, sleep or lose sight of her patient. Now, if this be true, how can one nurse keep herself awake and fresh night and day, for say even one week? Well, she cannot do it and do her work as it ought to be done, if she has been shut up in the sick room all day and goes to bed in it at night; it may so happen in a chance manner that all goes well, and it may also happen that all goes wrong; the patient in her sleep may turn on her side and so break down the wound by the stitches giving way, and ere she or the nurse awakes serious mischief takes place, that on awakening may be too far gone for remedying. Again, the fire may go out, and the temperature of the room alter so considerably as to affect and depress the patient, or retching may come on, and a sleepy nurse be too late in attendance to check it before the patient has moved herself, and in so doing moved dressings, if she has done no greater mischief; or worse ill still, while a tired out nurse is in a heavy sleep, may not hæmorrhage have done its deadly work e'er she awakes? Then let us be wise and simple in our calling. We are only poor weak mortals as our sister women; we need rest and sleep to a proper extent, and, to be healthy, air and a bath now and then, and no nurse ought to enter the room of such a patient who is not spotlessly clean in body and linen, and keep herself so by at least a daily wash all over, and this cannot always be done in her patient's room, nor is it nice for her to keep her box there, or even eat there; the latter is unwise in every way, both for patient and nurse, for meat and vegetables in the room of such a case must be enough to make the patient feel sick in her early days of illness, and when a little better she may long for food she must not have. I have nursed ovariotomies successfully, but have always been only too thankful to get the help of a sister in the profession, who took quite as much interest in the welfare of the patient as myself, and the patient has had the great benefit of having a bright, fresh, rested nurse to take the place of the tired one going off duty. Nurses must go on from year to year and keep their health or no one cares to employ them; and if a nurse wears herself out by foolishly doing double work at one case, well, the next case must suffer, for a nurse is but a woman and not a machine wound up to go for a certain time, and it is no use of her trying to be anything but what she is. She must season her ardour with the salt of common sense.

### Wants and Workers.

[The attention of correspondents is directed to the fact that "Helps in Sickness and to Health" (Scientific Press, 428, Strand) will enable them promptly to find the most suitable accommodation for difficult or special cases.]

Old linen of all kinds will be gratefully received by Sister Isabel, 43, Stepney Green, E., for the use of the sick poor in the district.

Linen is still required to stock the cupboards properly at the East End Mothers' Home, Commercial Road East. Sheets, pillow-cases, towels, and night gowns will be gladly acknowledged by Miss Blomfield, who is also glad of warm garments wherewith to supplement the scanty outfits provided by the poor mothers to take their babies home in.

Can you tell me of a home in or near London where a paralysed man could be received for £1 per week? Reply to "G. M.," care of the Editor, 428, Strand, W.C.

